# Biased estimates of clonal evolution and subclonal heterogeneity can arise from PCR duplicates in deep sequencing experiments

**DOI:** 10.1186/s13059-014-0420-4

**Published:** 2014-08-07

**Authors:** Erin N Smith, Kristen Jepsen, Mahdieh Khosroheidari, Laura Z Rassenti, Matteo D’Antonio, Emanuela M Ghia, Dennis A Carson, Catriona HM Jamieson, Thomas J Kipps, Kelly A Frazer

**Affiliations:** Moores UCSD Cancer Center, University of California San Diego, 9500 Gilman Drive, La Jolla, CA 92093 USA; Department of Pediatrics and Rady Children’s Hospital, University of California San Diego, 9500 Gilman Drive, La Jolla, CA 92093 USA; Clinical and Translational Research Institute, University of California San Diego, 9500 Gilman Drive, La Jolla, CA 92093 USA; Institute for Genomic Medicine, University of California San Diego, 9500 Gilman Drive, La Jolla, CA 92093 USA; Department of Medicine, University of California San Diego, 9500 Gilman Drive, La Jolla, CA 92093 USA; Stem Cell Program, University of California San Diego, La Jolla, CA USA

## Abstract

Accurate allele frequencies are important for measuring subclonal heterogeneity and clonal evolution. Deep-targeted sequencing data can contain PCR duplicates, inflating perceived read depth. Here we adapted the Illumina TruSeq Custom Amplicon kit to include single molecule tagging (SMT) and show that SMT-identified duplicates arise from PCR. We demonstrate that retention of PCR duplicate reads can imply clonal evolution when none exists, while their removal effectively controls the false positive rate. Additionally, PCR duplicates alter estimates of subclonal heterogeneity in tumor samples. Our method simplifies PCR duplicate identification and emphasizes their removal in studies of tumor heterogeneity and clonal evolution.

**Electronic supplementary material:**

The online version of this article (doi:10.1186/s13059-014-0420-4) contains supplementary material, which is available to authorized users.

## Background

Sequencing technologies have recently allowed for an unprecedented window into the process of cancer evolution [1]. Tumors are often heterogeneous with multiple subclones, which has become an important consideration in monitoring cancer evolution [2,3] and in choosing appropriate treatment regimens [4]. Deep-targeted sequencing [5] is frequently used in studying how these clones change over time. This technology has provided insights into subclonal phylogenetic structures in cancer [6] and mutational patterns that occur and are selected for during tumor progression [7–9] and in response to treatment [10]. Patient treatment can be informed by subclonal heterogeneity [11,12], and deep-targeted sequencing can be used to track recurrence and evolution by the sequencing of circulating tumor DNA [13].

Deep-targeted sequencing is well-suited to provide accurate frequency estimates because each read can provide independent information. However, obtaining accurate estimates of allele frequency can be complicated by the presence of PCR duplicates created during sequencing library preparation, yielding multiple copies of a single template that are each then sequenced. Duplicates will inflate the perceived sample size, for example, at a duplicate rate of 75%, for every 2,000 reads used to inform an allele frequency estimate, only 500 provide unique information. When comparing two samples, this inflated sample size could make it appear that there are significant differences when none exist. Biases in multi-template PCR can come from a variety of sources [14], including GC content [15,16]. Most frequently, PCR duplicates arise from a lack of DNA complexity due to low levels or quality of input DNA, or from biases in PCR. Exome sequencing studies at moderate (approximately 100X) depth rely on read position to identify potential PCR duplicates [17], but amplicon-based (molecular inversion probes [18], RainDrop Digital PCR (RainDance Technologies, Billerica, MA, USA), TruSeq Custom Amplicon (Illumina, San Diego, CA, USA)) methods commonly used for targeted sequencing have reads with the same start and stop positions. Hybridization-based methods, when sequenced deeply, can result in reads that are not PCR duplicates but have the same start stop locations by chance [19]. PCR-free methods are also available, but typically require higher amounts of DNA input (1 to 2 ug), limiting their use in cancer studies.

Single molecule tagging (SMT) is a method that has been developed to identify PCR duplicates using a DNA sequence tag [20–22] and has recently been incorporated into RNA sequencing [23]. Random oligomers are incorporated into a template prior to PCR amplification, and duplicates are tracked by the SMT oligomer. While this approach has been implemented in custom multiplex molecular inversion probe libraries [22], it requires computational expertise to implement and very large sample sizes to be cost effective. The ability to utilize SMT is not yet available in commercial kits for deep-targeted sequencing that are easier to design and more flexible.

Here, we adapt a commercially available custom amplicon-based deep sequencing kit to incorporate SMT and show that the SMT oligomer tags PCR duplicates. Using both experimentally derived and simulated duplicates, we test how the presence of duplicates affects estimates of clonal evolution and subclonal heterogeneity in tumor samples and show that the presence of PCR duplicates can inflate the false positive rate (FPR). Overall, our study provides a simple approach for removing PCR duplicates using an accessible deep-targeted sequencing kit and suggests that duplicates must be accounted for to obtain accurate frequency estimates important for studying heterogeneous populations, such as in studies of clonal evolution and cancer heterogeneity.

## Results and discussion

### Adapting Illumina TruSeq to use single molecule tagging

**Figure 1:**
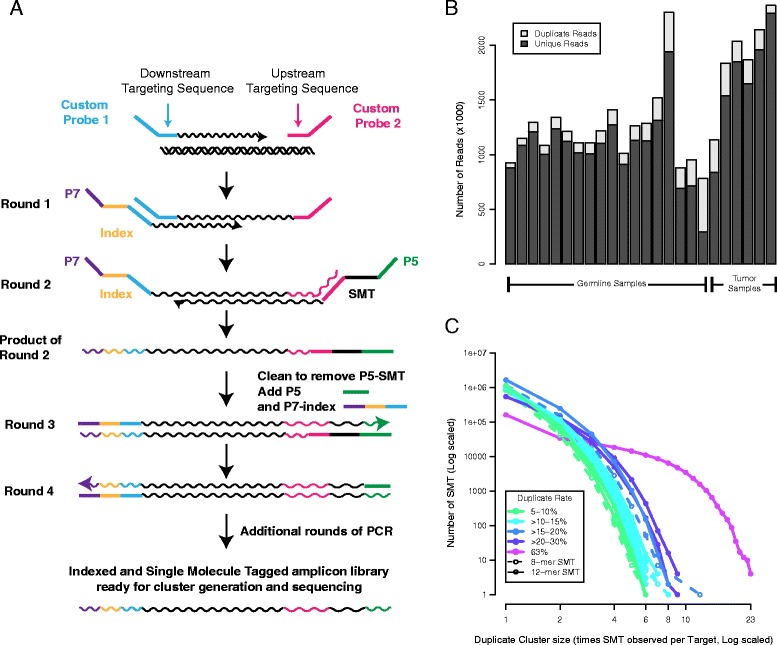
**Adaptation of Illumina TruSeq Custom Amplicon Kit to allow for single molecule tagging. (A)** Schematic of method showing amplification of target DNA using custom probes and flanking primers. The P5-SMT primer is the same as the standard P5 index primer, but contains a degenerate 12 N-mer sequence in place of the index. The incorporation of an Ampure Bead size selection step after two rounds of PCR removes unused P5-SMT, and the P5 primer is added to facilitate downstream amplification. Figure schematic is adapted from Illumina promotional material. **(B)** Stacked barplot showing number of paired-end reads, split into unique reads (dark grey) and SMT-identified duplicate reads (light-grey) in 24 samples (18 germline, 6 tumor). **(C)** For each of 18 germline samples, we show the number of SMTs by duplicate cluster size (the number of times that an SMT was observed at a given target within a sample). Higher overall duplicate rates within a sample were associated with larger duplicate clusters. Except for the sample with the highest duplicate rate, duplicate cluster sizes were generally less than 10. The length of the SMT (8- versus 12-mer) did not affect the distribution. SMT, single molecule tag.

The Illumina TruSeq Custom Amplicon Kit is a multiplex system for targeted sequencing that allows for approximately 1,500 amplicons to be sequenced at the same time. Custom probes with sequence flanking the target region are generated and, during sample preparation, the region is extended from one probe and then ligated to the second probe (Figure 1A). Next, during two rounds of synthesis, two different index primers are incorporated, generating individual double-stranded molecules that contain two indexes and flanking amplification sequences. Subsequent rounds of PCR amplify the molecules to create a library for sequencing. We adapted this kit to accommodate SMT by co-opting the second index sequence that is normally used for additional sample multiplexing. We incorporate a random 8- or 12-mer into the first round of amplification using a custom primer (P5-SMT) consisting of P5, an 8- or 12-mer SMT in place of the index, and a linker sequence. While this limits the number of samples that can be pooled together to 12, new i7 indexes or multiple P5-SMT primers could be used, containing a sample index followed by the N-mer. After two rounds, a single single-stranded product that has both P7 and P5 primer sequences is created. We then remove the remaining P5-SMT primer from the reaction and amplify the product with iP7 and P5. Thus, the SMT is copied into each subsequent PCR duplicate. During sequencing on either the MiSeq or HiSeq, the SMT oligomer is sequenced as an index read.

### Data generation

To test this method, we designed a custom amplicon library of 1,225 150-base-pair (bp) targets focused on heterozygous single nucleotide polymorphisms (SNPs) and somatic variants that were identified in Chronic Lymphocytic Leukemia (CLL) samples. We generated SMT-tagged deep-targeted sequencing data using two tumor samples sequenced at three different DNA inputs, and 18 germline DNA samples. Next, we sequenced to an average of 850X coverage using either an 8- or 12-mer SMT on the MiSeq, generating 150 bp paired-end reads (Additional file 1). We associated the resulting paired-end reads with an expected target by matching the first 22 bp of each read to the upstream and downstream targeting sequences of the expected targets (Figure 1A), and succeeded in finding a match for 96.6% of reads on average (Additional file 1). Unique reads were identified as the first read assigned to a target with an SMT that had not been seen for that target. Duplicate reads were those with SMT oligomers that had already been seen within a target. We observed duplicate rates ranging from 3% to 26% in the tumor samples and 5% to 63% (median 10%) in the germline samples (Additional file 1). Across the 24 samples, we observed a total of 29,183,611 unique reads and 4,050,234 duplicates (Figure 1B). Duplicate reads were widely distributed across different SMT oligomers and targeted sequences (Figure 1C). For samples with low duplicate rates, the SMT duplicate cluster sizes were small, with a single SMT not generally seen more than 10 times (Figure 1C). Samples with higher duplicate rates had concordant increases in duplicate cluster sizes. The largest SMT duplicate cluster contained 23 reads and was observed in the sample with the highest duplicate rate (Figure 1C). Within targets, SMT sequence diversity was high, differing from each other by 5 bp on average for 8 bp SMTs and 8 bp on average for 12 bp SMTs. Less than 0.1% of SMT pairwise comparisons differed by 1 bp, suggesting that the diversity is robust to sequencing errors. This indicates that our method effectively incorporates diverse SMT oligomers into the library preparation process and suggests that the Illumina TruSeq Custom Amplicon Kit generates a moderate number of PCR duplicate reads that are evenly distributed across targeted sequences.

### Single molecule tag-identified duplicates are PCR duplicates

While the expected complexity of a 12 bp random oligomer is high (4^12^ combinations), the same oligomer could be incorporated multiple times by chance if there are biases in how the oligomer was generated or incorporated. To show that SMT-identified duplicates are PCR duplicates, we first examine whether they are higher under conditions expected to produce a greater number of PCR duplicates, such as low DNA input, high GC sequence content, and in targets with relatively higher read depth. We then examine SMT sequence usage patterns and consistency of allele calls to determine how often SMT-identified duplicates are likely the result of PCR duplicates.

**Figure 2:**
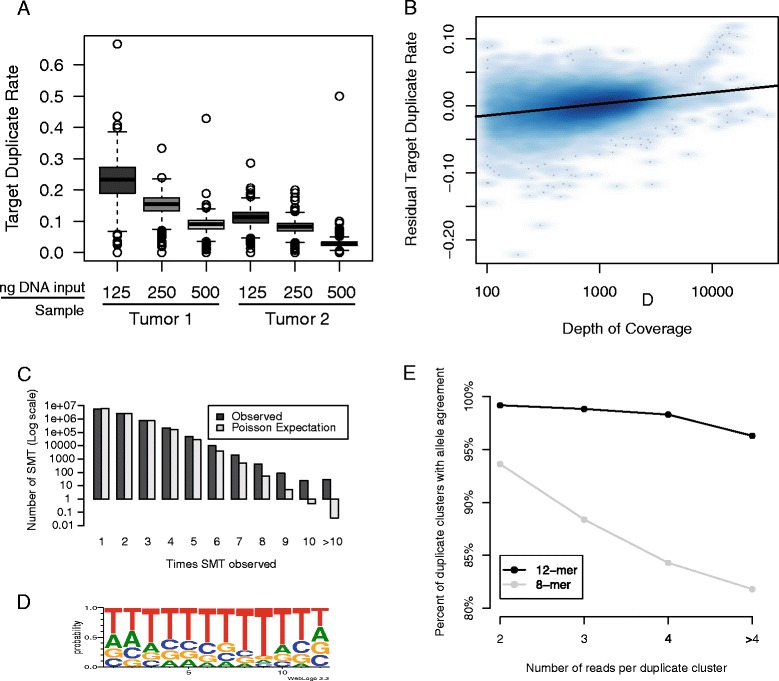
**Single molecule tag-identified duplicates represent PCR duplicates. (A)** Boxplot of target duplicate rate as a function of DNA input. DNA samples from two tumors were diluted and single molecule tag (SMT)-libraries were prepared. Duplicate rate, as identified by SMT sequence, was higher in the lower input DNA samples, consistent with lower starting complexity. **(B)** Smoothed scatterplot of the relationship between target duplicate rate, adjusted for sample-specific effects and GC content of insert and primers, and depth of coverage (shown log scale). **(C)** Barplot of the number of times that independent SMTs (one SMT per target per sample) were seen across targets and samples (black) compared to expectations by Poisson sampling (grey). **(D)** Motif identified using the 54 SMT sequences observed 10 or more times across all 14 12-mer germline samples and targets. **(E)** Agreement of allele calls within duplicate clusters for 12-mer and 8-mer SMT sequences. Percent allele call agreement is the percentage of duplicate clusters where all allele calls at the SNP of interest were consistent.

To test whether low DNA input was associated with higher SMT-duplicates, we analyzed two tumor samples at three different dilutions and then sequenced each to approximately 1,500X depth. For both tumors, we observed that lower input amounts of DNA were associated with higher rates of SMT-identified duplicates (Figure 2A). While the manufacturer recommended amount of input was 250 ng, we observed lower duplicate rates (6% to 7% point decrease) at 500 ng and used this amount for the 18 germline samples. We then examined whether the proportion of SMT duplicates in a given target was associated with GC content or, as a general indicator of PCR bias, depth of sequencing. We observed that target duplicate rate was correlated across samples (average pairwise r = 0.53). After adjusting for sample-specific effects in a multivariate model, we observed that duplicate rate was associated with coverage (Figure 2B, 0.5% increase in duplicate rate per 1,000X, *P* = 2 × 10^−274^); the GC content of the insert (0.6% increase in duplicate rate per 10% increase in GC content, *P* = 7 × 10^−184^); and GC content of the upstream targeting primer (0.1% decrease in duplicate rate with 10% increase in GC content, *P* = 7 × 10^−8^) and of the downstream targeting primer (0.06% decrease in duplicate rate with 10% increase in GC content, *P* = 6 × 10^−4^). These results show that SMT-duplicates increase at low input DNA levels and are associated with other factors that affect PCR duplication rates.

To estimate the proportion of reads within a target that are likely to be duplicate because of random incorporation of the same sequence, we examined the distribution of SMT-sequence usage. While SMT usage within a target may be due to PCR duplicates, SMT usage across targets and across samples will not be. We therefore analyzed SMT usage across all unique SMTs using one instance of each 12 bp SMT per target per sample, for a total of 14,485,830 unique SMTs from the 14 germline samples that used a 12 bp SMT. The theoretical maximum number of times we could observe a single SMT sequence was 17,150 (14 samples × 1,225 amplicons). We calculated the number of times we would expect to see SMT sequences using the Poisson distribution assuming 4^12^ (16,777,216) possible SMTs, an equal probability of choosing each one, and 14,485,830 observations, and compared this to our observed distribution (Figure 2C). We observed a general similarity in distribution, with a bias towards seeing some SMTs more than expected. In total, we observed 9,438,051 SMT sequences, slightly lower than expected by Poisson sampling (9,701,993). Each was observed on average 1.53 times, and 54 were observed 10 or more times (range 10 to 915). We would not have expected to see more than one SMT sequence more than nine times if there was no sequence bias. Motif detection suggested overrepresented SMT sequences were enriched for a stretch of Ts (Figure 2D). To estimate the proportion of SMT that would be expected to be the same sequence by chance given this distribution of sequence bias, we sampled SMTs from the observed distribution of SMT to the read depth of unique reads of a typical sample (Sample Germ05, average unique target coverage 1,074), and counted the number of times that the same SMT was sampled within each target. We observed that 0.006% of reads were the same due to SMT usage, indicating that the observed bias is not relevant for deep sequencing at this depth. In this same sample, if we restrict analysis to the first 8 bp of the observed SMTs, we see 1.7% of the reads within a given target shared the same SMT by chance, suggesting that 8 bp does not have enough complexity to avoid sampling the same SMT multiple times at high read depth. These results indicate that while there is some preferential SMT usage associated with higher usage of thymidine bases, in general SMT usage is diverse and observed duplicate SMT sequences for a given target within a sample are unlikely to be due to preferential use of specific SMT sequences when the SMT length is 12 bp.

We then examined allele calls at SMT-duplicates to determine if they carry the same allele at heterozygous SNP loci. For each sample, we identified reads that overlapped an SNP and asked whether all the alleles at the SNP agreed within each SMT-duplicate cluster. For samples using a 12-mer SMT, we observed high agreement when the duplicate cluster consisted of two reads (227,941 out of 229,849 (99.2%)), as well as for larger clusters (Figure 2E). For those with an 8-mer SMT, the agreement was poorer (36,902 out of 39,415 (93.6%)), with agreement decreasing at higher duplicate cluster sizes. For both 8- and 12-mer SMTs, the discordance was not due to sequencing error as we observed very high concordance between both reads of paired-end reads (473,625 out of 473,633 (99.998%) for 8-mer and 2,425,721 out of 2,425,763 (99.998%) for 12-mer). This suggests that while 8-mers are not informative enough to fully discriminate PCR duplicates from random incorporation of the same SMT, 12-mers effectively identify PCR duplicates and thus we recommend the use of a 12-mer SMT for sequencing studies at depths of approximately 1,000X.

### The effect of duplicates on estimates of clonal evolution

To determine how duplicates affect estimates of clonal evolution, we have devised an approach where we do not expect any real differences between two samples, allowing us to study how the addition of duplicates - simulated or experimentally derived - affect the number of sites that look different between the two samples (FPR). From a single parent sample, we randomly sample reads to produce a pair of samples that arise from the same distribution. We add in either simulated or experimentally derived duplicates and test for differences between heterozygous SNPs between the samples. Under an alpha of 0.05, we expect that 5% of the tests would be significantly different, and can examine if the FPR increases relative to alpha as a function of duplicate rate.

**Figure 3:**
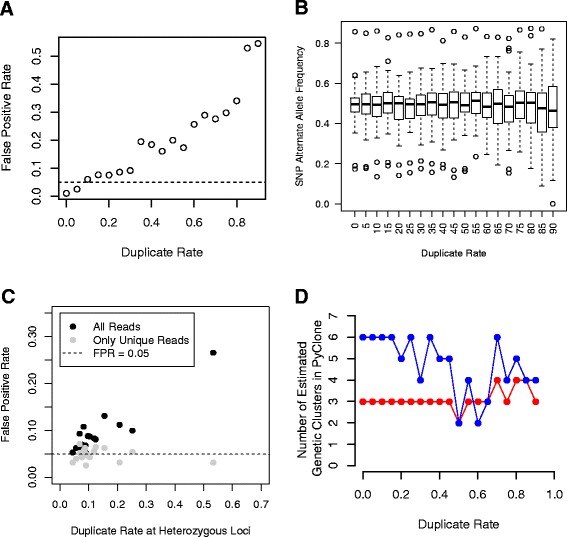
**PCR duplicates inflate the false positive rate of differences between samples and alter measures of clonal heterogeneity. (A)** False positive rate (FPR) for tests at heterozygous single nucleotide polymorphisms (SNPs) between groups of reads randomly allocated from the same sample with a varying percentage of simulated duplicates. Dotted line indicates FPR = 0.05. **(B)** Boxplot indicating higher variability in alternate allele frequency at heterozygous SNPs as duplication rate increases. **(C)** Increase in FPR for tests at heterozygous SNPs when single molecule tag-duplicates are present (black) or removed (grey). Dotted line indicates FPR = 0.05. **(D)** Estimates of the number of genetic clusters in two tumor samples (in red and blue respectively) becomes variable as duplicate rate increases. The number of clusters was calculated using PyClone.

To examine the full range of effects across duplicate rates, we initially simulated duplicates. We randomly sampled unique reads from a single germline sample to create a pair of samples. Within each of these samples we then sampled from the unique reads to simulate varying proportions of duplicate reads under a constant read depth, and tested for differences in allele frequencies at heterozygous SNPs between the paired samples using a Fisher’s exact test. We observed that as the percentage of duplicates increased, the proportion of SNPs that were called significant at an alpha of 0.05 also increased (Figure 3A). This is consistent with an increase in variance of the alternate allele frequency estimate at higher duplicate rates, even though the total number of reads is the same (Figure 3B).

We applied this approach to experimentally identified duplicate reads in the 18 germline DNA samples, and compared the FPR when only unique reads were used to that observed when duplicates were included. At the heterozygous SNPs identified in each sample, SMT duplicate rates varied by 4% to 53%, with most showing rates between 5% and 25%. For each sample, unique reads were identified by SMT sequence, and randomly split into two paired samples. Duplicate reads were linked to unique reads by SMT sequence and allocated to the appropriate sample. We then tested for allele frequency differences between the paired samples at heterozygous SNPs using a Fisher’s exact test. We observed a strong correlation between overall duplicate rate and the FPR when duplicates were included, with the highest duplicate rate showing over 25% SNPs different between the samples (Figure 3C). When duplicates were removed, however, the FPR dropped to approximately 5% across all paired sample comparisons, effectively controlling the FPR to the alpha level. In all samples, inclusion of duplicate reads increased the FPR. Thus, even at low levels, inclusion of PCR duplicates inflates the FPR, and identification and removal of PCR duplicates by SMT appropriately controls the FPR. These results indicate that when using read depth to test for changes in allele frequency during clonal evolution, removal of PCR duplicates is required to avoid false positives.

To show that the duplicate rate was not affected by the SMT labeling process, we analyzed an unrelated dataset that was generated using an unmodified Illumina Truseq Custom Amplicon kit. This dataset containing 87 germline and cancer DNA samples [24] from breast cancer patients had been run on the same target set twice. At sites with at least 50X coverage, we tested whether heterozygous SNPs were different between the two replicate samples, using a Fisher’s exact test. Overall, we observed that 11.8% (865/7338) of SNPs showed a difference (P < 0.05) between duplicates, with 93% of samples showing a FPR > 5% (range 0 – 30%, Additional file 2). These rates are similar to those observed in the SMT-labeled samples and suggest that unaccounted duplicates are present using the unmodified TruSeq protocol.

To assess how quantification of clonal heterogeneity is affected by the presence of duplicates, we examined two chronic lymphocytic leukemia samples from the same patient collected after diagnosis or prior to treatment that respectively had 9% and 3% duplicate rates. We analyzed our deep sequencing data at 16 somatic loci identified through exome sequencing as mutated in this tumor. We compared the number of genetic clusters estimated using PyClone [25] before and after removing SMT-identified duplicates and observed no difference, suggesting that this approach may be robust to low numbers of duplicates. To investigate how estimation of clonal heterogeneity is affected by high duplicate rates, we modeled duplicates across a range of proportions by first removing SMT-identified duplicates and then randomly sampling from the unique reads to create defined proportions of duplicates (0% to 90%). We estimated the number of genetic clusters as a function of duplicate rate and show that the number of genetic clusters becomes much more variable at increased duplicate rates for both samples (Figure 3D), especially at high (>50%) duplicate rates. These results indicate that the presence of high levels of duplicates can artificially inflate or deflate separation of genetic clusters, resulting in altered estimates of clonal heterogeneity.

## Conclusions

PCR duplicates can be unacknowledged in targeted sequencing studies because the tools to identify duplicates in accessible commercial kits are poor. Our study demonstrates the importance of identifying and removing PCR duplicates in studies of clonal evolution and cancer heterogeneity and provides a simple modification to a commercially available kit that allows for effective identification of PCR duplicates in deep-targeted sequencing. We show that the presence of duplicates can inflate the FPR when testing for changes in allele frequency between two samples and that estimates of clonal heterogeneity can be much more variable in the presence of duplicate reads. When there is strong evidence for clonal evolution by the presence of new variants at high frequency or large changes in allele frequency (>20%), the presence of moderate levels of PCR duplicates (<25%) is not likely to be an issue. However, duplicates may cause false interpretations in the cases of unknown high duplicate rates or no clonal evolution.

While we focus on a single kit, PCR duplicates are present in many sequencing applications. Targeted microdroplet methods, such as RainDance, will have a limited number of original fragments based on the number of microdroplets and the number of targets, creating a ceiling for complexity. Methods that fragment prior to hybridization can be filtered using start and stop positions, but can also contain unidentified duplicates due to slight differences in alignment. The issues that we discuss are relevant to these approaches as well, and suggest that they would benefit from the incorporation of SMT tagging.

Removal of PCR duplicates will be relevant for a number of applications that use targeted sequencing. Metastatic cancer can now be monitored through deep sequencing of circulating tumor DNA [13], and the potential for poor DNA quality combined with deep sequencing requirements make PCR duplicates an important concern, especially if the results impact clinical decisions. Further, methods to study highly diverse mixtures like the immune repertoire [26–28] or viral heterogeneity [29] rely on accurate read depths to infer population dynamics. Our approach can also be applied to applications that require high accuracy of rare variant calls, such as in pooled populations or cancer, where SMTs have been used to group duplicate reads together in order to cancel out sequencing errors and obtain highly accurate variant calls [22]. Thus, while we focus on a clonal evolution and heterogeneity using a single commercial kit, our results have general implications for a wide variety of study designs and sequencing methods.

## Materials and methods

### Sample collection

Samples were chosen from participants of the CLL Research Consortium. The University of California San Diego Institutional Review Board approved the study and all participants gave informed consent. DNA was isolated from tumor samples using the AllPrep DNA/RNA Mini kit (Qiagen®, Valencia, CA, USA) according to the manufacturer’s instructions. Concentrations were determined by fluorometry (Qubit®, Life Technologies). Saliva DNA was isolated using an Oragene kit (DNA Genotek, Kanata, Ontario, Canada). Sixty-two of the breast cancer and germline samples have been previously described [24]. The other 25 samples were retrieved from the University of California, San Diego Biorepository at Moores Cancer Center, and processed as previously described [24].

### Targeted resequencing of somatic variants

We performed deep-targeted sequencing using the Illumina TruSeq Custom Amplicon kit. Using DesignStudio software, probes were successfully designed to cover approximately 1,000 somatic variants identified in an exome sequencing project of 18 CLL patients to study clonal evolution (in preparation) and about 200 common SNPs, resulting in 1,225 targets of around 150 bp in length. To account for PCR duplicates, we incorporated a novel modification of the kit that introduced an SMT into each read by modifying the i5 index to encode a random N-mer of 12 bases. Sequencing libraries were prepared following the TruSeq Custom Amplicon Library Preparation Guide with the following modifications. For most samples, 500 ng of DNA was used, except for when dilution experiments where performed and 125, 250, or 500 ng was used. Attempts to create libraries using less DNA (62 ng or 16 ng) did not succeed. Extension-ligation was performed according to manufacturer’s protocol. A two-step modified PCR amplification was performed. In the first round, we used the i7 primer supplied with from the TruSeq Custom Amplicon Index Kit and a custom primer, P5-SMT (5′AATGATACGGCGACCACCGAGATCTACACNNNNNNNNNNNNACACTCTTTCCCTACACGACGCTCTTCCGATCT3′), in place of the i5 primer and at the same concentration as the i5 primer. Two PCR cycles were performed as follows: 95°C 3 minutes, followed by 2 cycles of 95°C for 30 seconds, 66°C for 30 seconds, and 72°C for 60 seconds. Following two cycles of PCR, Ampure Bead cleanup was carried out according to standard protocol and eluted in 25 uL. Following cleanup, 20 uL of PCR product, 22 uL of PMM2/TDP1, 4 uL of the same i7 primer supplied with the TruSeq Custom Amplicon Index Kit, and 4 uL of a 6.25 μM custom primer, TruSeqP5 (5′ AATGATACGGCGACCACCGAGATCTACAC 3′) were added to PCR tubes, and PCR was carried out using the following conditions: 95°C for 30 seconds; 22 cycles of 95°C for 30 seconds, 66°C for 30 seconds, and 72°C for 60 seconds; and finally 72°C for 5 minutes (note that number of cycles is specific to TruSeq Custom Amplicon Design). Ampure bead cleanup was performed, library quality was assessed on an Agilent Bioanalzyer (Agilent Santa Clara, CA, USA) using a DNA 1000 chip, and concentration determined by Qubit and quantitative PCR using the KAPA Library Quantification Kit (Kapa Biosystems, Woburn, MA, USA). Samples were pooled based on Qubit determined concentrations and 12 samples per run sequenced at 12 pM using Illumina MiSeq V2 sequencing reagents and the following run set up: Read 1: 151 cycles; Read 2: 8 cycles; Read 3: 12 cycles (or 8 cycles); Read 4: 151 cycles. Note that library normalization using library normalization beads cannot be used with this modified protocol.

The samples from patients with breast cancer were processed following the same pipeline. They were sequenced using Illumina MiSeq V2 sequencing reagents with a different set up: Read 1: 301 cycles; Read 2: 8 cycles; Read 3: 8 cycles; Read 4: 301 cycles.

#### Read processing and filtering

To obtain reads with SMT information in the read names, FASTQ files were generated using CASAVA (v1.8.2). A random index was listed as the second index read in the sample sheet and bcl files were converted to FASTQ files using configureBclToFastq.pl. Reads were retrieved from the unaligned folder. To clean the reads of poor quality data and to link each read to its target, read pairs were assigned to their respective targets by matching the first 22 bp of Read 1 to the reverse complement of the downstream locus-specific oligo custom primer sequence and the first 22 bp of the Read 2 to the upstream locus-specific oligo custom primer sequence, allowing for two differences (Levenstein distance). Read pairs were kept when both Read 1 and Read 2 matched the same target (approximately 95% of reads). Read pairs for each target were grouped and trimmed of their appropriate primer sequences using cutadapt (settings) [30], resulting in read lengths of 122 to 130 bp. Within a given target, unique reads corresponded to the first time an SMT was used within that target and all subsequent uses of the SMT were duplicate reads. The resulting reads (>35 bp long) were aligned to the genome using the BWA-MEM [31] algorithm with default settings. FASTQ files from the 87 breast cancer and germline samples were processed using the same pipeline, except for the SMT sequence.

#### *Variant calling*

Variants were called in each sample using GATK UnifiedGenotyper [32]. Somatic variants were identified by genotyping tumor and germline samples from the same patient together, and testing for differences in allele frequencies across samples using a 2 × 3 Fisher’s exact test. Allele counts were calculated from the Allele Count variable (AC) in GATK, divided by two to account for the SNP being sequenced twice within a paired read. Sites that reached an average coverage of 50X in all samples from a given patient and a variant quality (QUAL) score of 50 were used to identify somatic variants. Sites were considered somatic if the germline alternate frequency was <10% and a 2 × 3 Fisher’s exact test of allele counts was significant (Benjamini-Hochberg false discovery rate <0.05). Heterozygous SNPs were identified in each germline sample separately, using unique reads only. SNPs that were previously identified in the short genetic variation database (dbSNP build 135) and that reached a QUAL score of at least 100 were used. The 87 breast cancer and germline samples were processed in the same manner, except only the first read of the paired-end read was used in variant calling.

#### Single molecule tag sequence usage

The number of times that we would expect to see each SMT sequence was calculated using the Poisson distribution (dpois in R). The probability was calculated for a given number of observations using a lamba of the probability of observing each sequence (1/4^12^) times the number of samples (14,485,830), multiplied by the number of possible SMTs (4^12^). Motif enrichment was characterized using WebLogo v.3 [33,34].

#### Concordance at single molecule tag duplicates

To determine if duplicate reads carried the same allele, for each sample, heterozygous SNPs were identified using GATK and all unique reads. Then, targets were identified that overlapped each SNP and the corresponding reads were analyzed for the SNP alleles. Only Read 1 was used. SMTs that occurred twice were identified for each target and the allele at the expected SNP position was identified by the location in the sequence. If the pair of reads with the same SMT showed the same allele, it was considered concordant, whereas if they showed a different allele, they were considered discordant. SNPs that reported an allele other than the alternate or reference allele more than 10% of the time were excluded from the analysis. To calculate paired read concordance, Read 1 and Read 2 of the paired-end read were compared.

#### Simulation of duplicate reads within a sample

A single sample (Germ10) was split into two groups of reads (approximately 145,000 paired reads each) using *sample* in R. For each duplicate rate (0% to 90%), unique reads were sampled from each group of reads (that is, 72,500 reads for 50%) and then duplicates were generated from this group of reads by random sampling and replacement. Reads were simulated similarly for tumor samples that were collected from the same patient after diagnosis or prior to treatment, but the reads were not split into two groups. Within a single sample, unique reads were identified and a subset (1 - duplicate rate) was randomly chosen. Duplicate reads were then sampled with replacement from the chosen unique reads.

#### Testing for differences at heterozygous loci

Heterozygous loci were identified using all unique reads for each sample using GATK. Each pair of samples (either with simulated duplicates or with/without experimental duplicates) was genotyped at these loci using GATK UnifiedGenotyper (−−genotyping_mode GENOTYPE_GIVEN_ALLELES). Allele counts were calculated from the Allele Count variable (AC) in GATK, divided by two. Differences between the pools were identified at each locus using a 2 × 2 Fisher’s exact test (fisher.test in R) of allele counts and associations at *P* <0.05 were called significant.

#### *PyClone*

PyClone (v.0.12.3) was downloaded [35] and used to estimate the number of clones in tumor samples from each patient. Copy number estimates were made from Illumina HumanOmni2.5 BeadChip data using CNVPartition, but none were identified overlapping the somatic loci for this patient. For duplication rates of 0% to 90%, a single BAM file containing a subset of the unique reads and duplicate reads to make up the remainder read depth was generated for each tumor sample. Genotypes were called using GATK and allele counts were calculated from AC, divided by two. Each pair of samples was run separately in PyClone using default or suggested settings. The number of genetic clusters identified for each tumor sample at various duplication rates was reported.

### Data access

Sequence data is available through dbGaP study ID phs000767 [36].

## Additional files

Additional file 1:
**Table listing sequencing statistics.**
Additional file 2:
**Barplot showing the percentage of SNPs different between replicate samples.**

## Abbreviations

AC: Allele Count variable
bp: base pair
CLL: Chronic Lymphocytic Leukemia
FPR: false positive rate
SMT: single molecule tag
SNP: single nucleotide polymorphism
