# Supplementary figures and images for: Biased estimates of clonal evolution and subclonal heterogeneity can arise from PCR duplicates in deep sequencing experiments
Source: Genome Biol. 2014 Aug 7;15(7):420. doi: 10.1186/s13059-014-0420-4 (PMC4165357; doi:10.1186/s13059-014-0420-4)

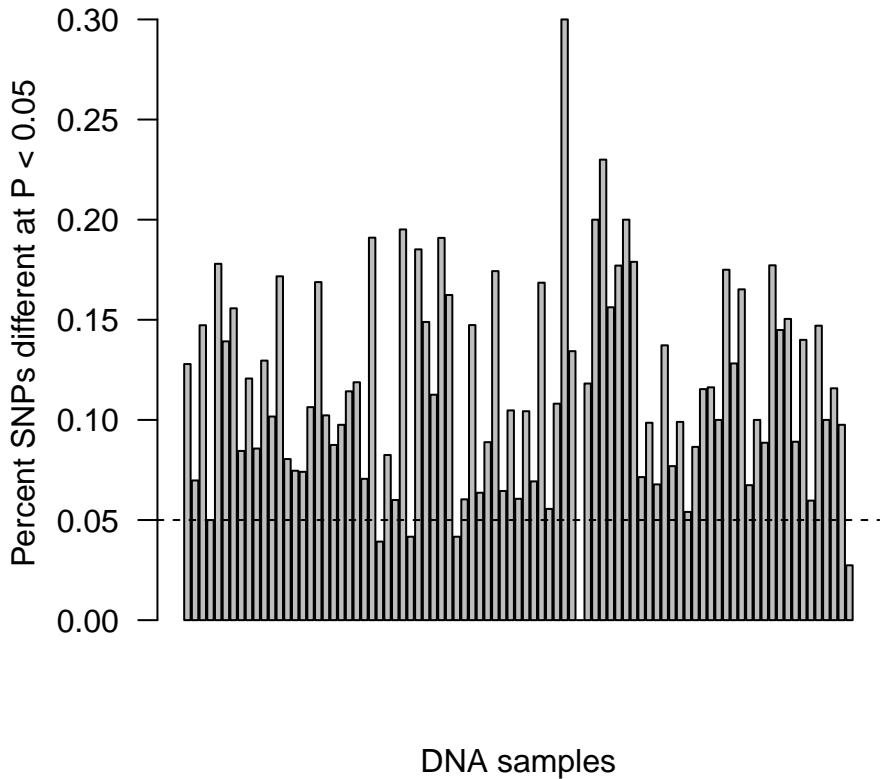

Supplement: Additional file 2: — Barplot showing the percentage of SNPs different between replicate samples. [file 13059_2014_420_MOESM2_ESM.pdf]
